# Supplementary material for: Genome Characterization of Bacteriophage KPP-1, a Novel Member in the Subfamily Vequintavirinae, and Use of Its Endolysin for the Lysis of Multidrug-Resistant Klebsiella variicola In Vitro
Source: Microorganisms. 2023 Jan 13;11(1):207. doi: 10.3390/microorganisms11010207 (PMC9862379; doi:10.3390/microorganisms11010207)
Supplement: Supplementary file 1 [file microorganisms-11-00207-s001.zip › Annex 2.pdf]

Annex 2: Assessment of KPP-1 lysis efficacy on two *Klebsiella variicola* isolates

- Strains: KPP-1 phage stock, bacterial strains: propagation host (Lab isolate 1; Lab stock #200) and lab isolate 2 (Lab stock #229).
- Buffer: SM buffer (5 mM Tris-HCl, 100 mM NaCl, 10 mM $MgSO_4$, pH 7.4)
- KPP-1 phage titer was determined by plaque counting.
- The average titer of two trials were 1.0 x 10^{10} pfu/mL for both *Klebsiella* isolates (Lab stock #200 and 229)

KPP-1 phage activity confirmation

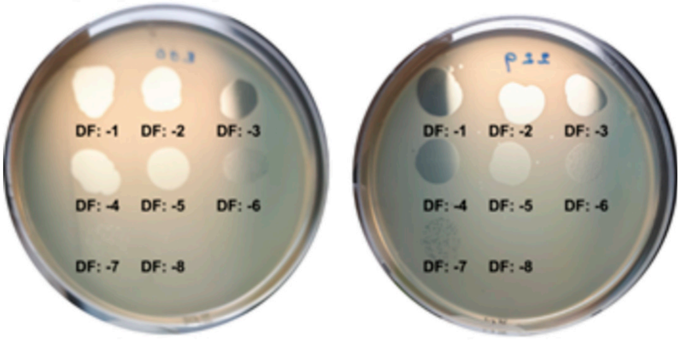

**Lab stock #200**	**Lab stock #229**
**Phage titer 1.0 x 10^{10} pfu/mL**	**Phage titer 1.0 x 10^{10} pfu/mL**

Trial	Lab isolate 1 (Stock# 200) (Propagation host)	Lab isolate 2 (Stock# 229)
1	1.0 X 10^{10} pfu/mL	1.2 X x10^{10} pfu/mL
2	1.0 X 10^{10} pfu/mL	0.8 X x10^{10} pfu/mL

Step 1: Plate elution

- Strain: KPP-1 and bacterium *Klebsiella* isolates (stock # 200 & 229).
- Method: Plate elution
  - Make 1% bacteria inoculum using O/N culture broth. When the OD_{595} reaches 0.6, add 100 µL of bacteria into 4 mL of TA–soft agar medium. Then add 100 µL of phage stock into the same TA–soft agar medium and overlay the mixture on pre-solidified NB plates. Incubate the plate for 10 h at 37 °C. Collect the lysate by adding 5 mL of SM buffer.

Step 2: Liquid culture

- ❑ Prepare TA broth (500 mL) and make 1% inoculum using N/N culture of propagation strain (stock#200). Incubate the culture at 37 ºC with agitation until the OD_{595} just reach 0.18 (<0.2).
- ❑ Inoculate filter sterilized phage stock at 0.1 MOI. Keep the bacteria and phage mixture on the benchtop for 10 min to facilitate adsorption and allow it to incubate with slow agitation (100 rpm).
- ❑ Stop the incubation when clear lysis is observed and collect the supernatant by centrifugation followed by filter sterilization
- ❑ Determine the phage titer and use it for PEG precipitation.

Step 3: Polyethylene glycol (PEG) precipitation

- ❑ Add polyethylene glycol 8000 (Sigma), 10% (w/v) and NaCl 5% (w/v) into phage filtrate and mix well in plastic centrifuge bottles.
- ❑ Keep overnight at 4 ºC.
- ❑ Centrifuge at 8000 x g for 30 min at 4 ºC (use angled rotor).
- ❑ Carefully remove centrifuge bottles from the centrifuge rotter and observe the white color precipitate at the walls of the bottles (opposite the wall of the rotor axis).
- ❑ Remove the supernatant and collect the precipitate by forceful flushing the wall with an appropriate volume of SM buffer.
- ❑ Determine the phage titer and dialyze against the SM buffer.
- ❑ Use dialyzed phage particles for downstream studies.
