# Supplementary material for: Genome Characterization of Bacteriophage KPP-1, a Novel Member in the Subfamily Vequintavirinae, and Use of Its Endolysin for the Lysis of Multidrug-Resistant Klebsiella variicola In Vitro
Source: Microorganisms. 2023 Jan 13;11(1):207. doi: 10.3390/microorganisms11010207 (PMC9862379; doi:10.3390/microorganisms11010207)
Supplement: Supplementary file 1 [file microorganisms-11-00207-s001.zip › Supplementary Figures S1-S3.pdf]

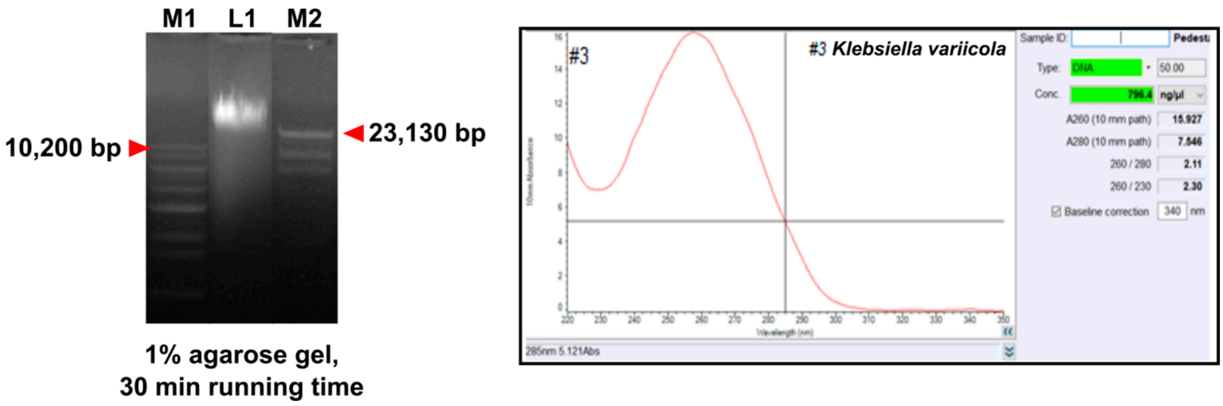

Supplementary Figure S1. The quality assessment of genomic DNA preparations. The quality assessment of KPP-1 genomic DNA isolation was done by agarose gel electrophoresis. M1 indicates a 1 kb marker (Bioneer, Republic of Korea), L1 indicates KPP-1 genomic DNA, and M2- indicates the Lamda DNA/Hind III marker (Bioneer, Republic of Korea).

PhageTerm analysis

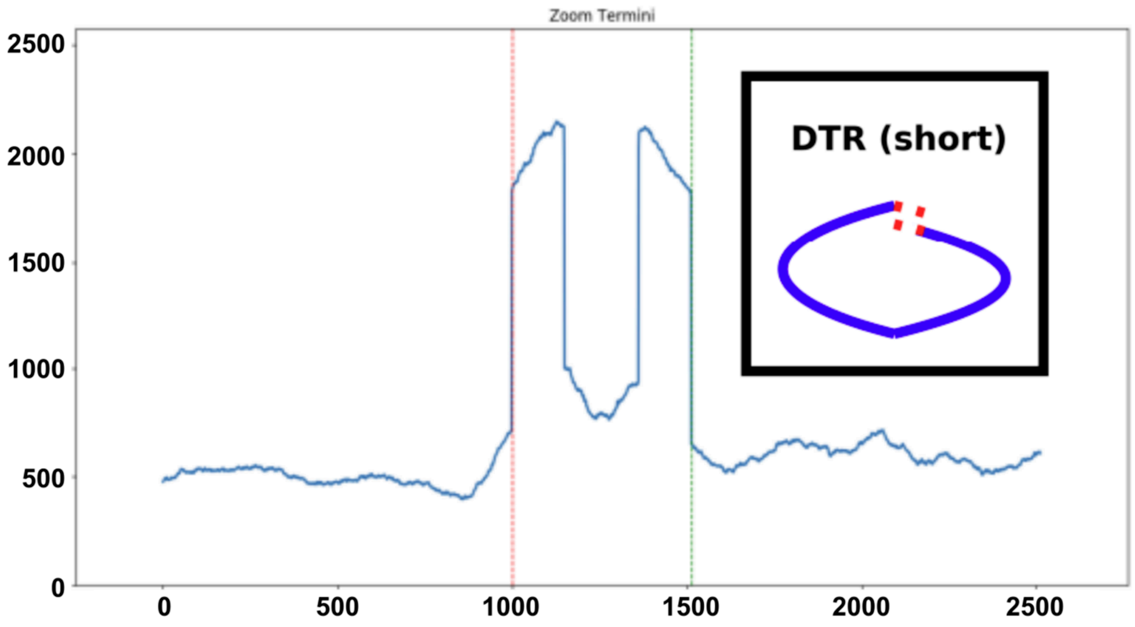

**Direct Terminal Repeats: 514 bp*

Strand	Location	SPC	R
+	116316	1115	51.0
	116372	22	-
	116378	19	-
	116365	14	-
	31802	13	-
-	116829	1171	56.0
	116768	21	-
	116619	15	-
	116711	14	-
	116708	13	-

SPC

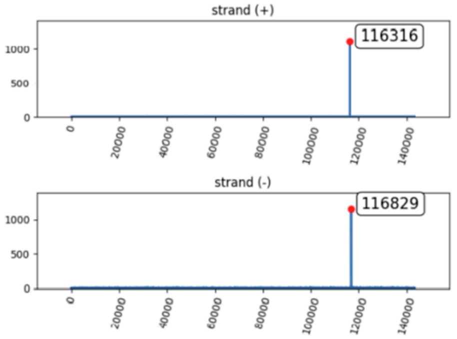

Supplementary Figure S2. The KPP-1 genome packaging strategy. The KPP-1's genome packaging strategy was investigated using PhageTerm software. The total number of raw Fastq reads was aligned against the assembled KPP-1 fasta sequence. A 514 bp long direct terminal repeats and their positions could be discovered. The phage packaging strategy was determined to be based on short DTR (Direct Terminal Repeats) similar to T7 bacteriophages.

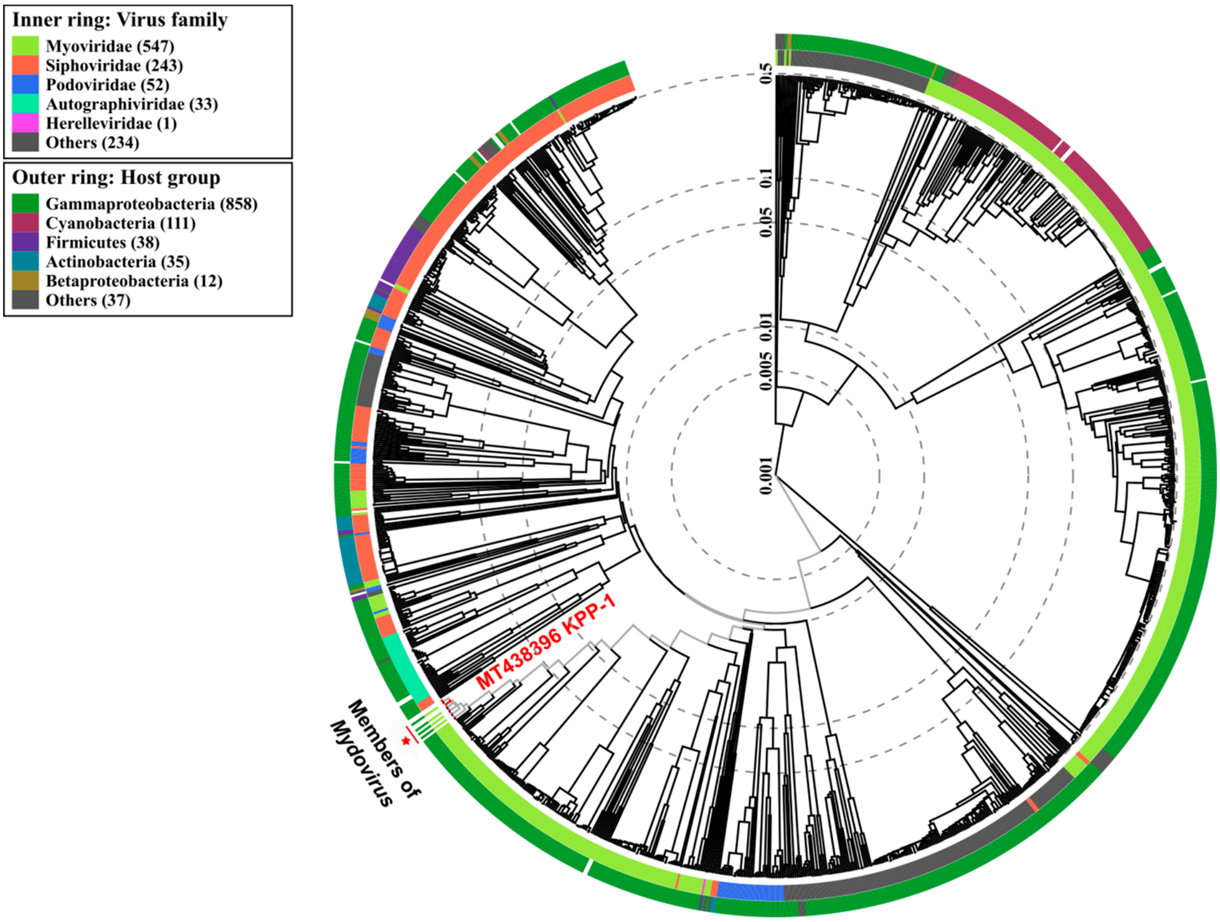

Supplementary Figure S3. VipTree analysis. The KPP-1 proteome was compared against the comprehensive proteomic analysis tool VipTree web server. The circular phylogenetic tool demonstrates the KPP-1 proteome relationship to a large collection of bacteriophages and their host bacterium proteomes. Red stars indicate the relative location of *Mydovirus* bacteriophages. The red color branch indicates the position of KPP-1 in the large pool of bacteriophage proteome.
