# Supplementary material for: Genome Characterization of Bacteriophage KPP-1, a Novel Member in the Subfamily Vequintavirinae, and Use of Its Endolysin for the Lysis of Multidrug-Resistant Klebsiella variicola In Vitro
Source: Microorganisms. 2023 Jan 13;11(1):207. doi: 10.3390/microorganisms11010207 (PMC9862379; doi:10.3390/microorganisms11010207)
Supplement: Supplementary file 1 [file microorganisms-11-00207-s001.zip › Supplementary Table S1.pdf]

Supplementary Table S1. Host range analysis of Klebsiella phage KPP-1

Lab Ref. No	Bacterium	Medium	Growth Temp. °C	Lytic ability	Origin
200	*Klebsiella variicola*	NB	37	++++	Propagation host (Laboratory isolate)
229	*Klebsiella variicola*	NB	37	++++	Laboratory isolate
228	*Klebsiella pneumoniae*	NB	37	-	,,
231	*Klebsiella pneumoniae*	NB	37	-	,,
170	*Aeromonas hydrophila*	TSB	25	-	,,
120	*Aeromonas salmonicida*	TSB	25	-	,,
226	*Aeromonas sobria*	TSB	25	-	,,
192	*Aeromonas veronii*	TSB	25	-	,,
194	*Aeromonas veronii*	TSB	25	-	,,
97	*Cronobacter sakazakii* ATCC29544	LB	37	-	ATCC
203	*Edwardsiella piscicida*	BHI	25	-	Laboratory isolate
204	*Edwardsiella piscicida*	BHI	25	-	Laboratory isolate
175	*Enterobacter cloacae*	LB	37	-	Laboratory isolate
25	*Enterococcus faecalis*	LB	37	-	Laboratory isolate
96	*Escherichia coli* O157:H7 ATCC35150	LB	37	-	ATCC
225	*Morganella morganii*	NB	37	-	Laboratory isolate
182	*Pantoea dispersa*	NB	37	-	,,
324	*Providencia rettgeri*	TSB	37	-	,,
14	*Shigella flexneri*	LB	37	-	,,
38	*Shigella flexneri*	LB	37	-	,,
146	*Staphylococcus aureus*	TSB	37	-	,,
326	*Staphylococcus haemolyticus*	TSB	37	-	,,
59	*Listeria monocytogenes*	TSB	37	-	,,
206	*Vibrio tubiashi*	BHI+ 1% NaCl	25	-	,,
207	*Vibrio tubiashi*	BHI +1% NaCl	25	-	,,
209	*Vibrio crassostrea*	BHI +1% NaCl	25	-	,,
210	*Vibrio crassostrea*	BHI +1% NaCl	25	-	,,
212	*Vibrio splendidus*	BHI +1% NaCl	25	-	,,
213	*Vibrio splendidus*	BHI +1% NaCl	25	-	,,
214	*Vibrio ichthyonteri*	BHI +1% NaCl	25	-	,,
215	*Vibrio ichthyonteri*	BHI +1% NaCl	25	-	,,
220	*Vibrio cyclitrophicus*	BHI +1% NaCl	25	-	,,
