# Supplementary material for: Genome Characterization of Bacteriophage KPP-1, a Novel Member in the Subfamily Vequintavirinae, and Use of Its Endolysin for the Lysis of Multidrug-Resistant Klebsiella variicola In Vitro
Source: Microorganisms. 2023 Jan 13;11(1):207. doi: 10.3390/microorganisms11010207 (PMC9862379; doi:10.3390/microorganisms11010207)
Supplement: Supplementary file 1 [file microorganisms-11-00207-s001.zip › Supplementary Table S2.pdf]

Supplementary Table S2. Functional grouping of ORFs in the genome of *Klebsiella* phage KPP-1

Functional group	Putative function	Locus tag	Homologous organism	Percent sequence identity (%)
Structural	Putative tail fiber protein	KPP_1411	Klebsiella phage vB_KpnM_KB57	100
	Putative membrane protein	KPP_1441	Klebsiella phage vB_KpnM_KB57	100
	Putative tail fiber protein	KPP_1461	Proteus phage Mydo	100
	Tail length tape-measure protein	KPP_1591	Klebsiella phage vB_KpnM_KB57	96.63
	Structural protein	KPP_1631	Klebsiella phage vB_KpnM_KB57	99.8
Nucleotide metabolism and transcription	Nicotinamide phosphoribosyltransferase (EC 2.4.2.12)	KPP_11111	Proteus phage Mydo	100
	Ribose-phosphate pyrophosphokinase (EC 2.7.6.1)	KPP_11121	Klebsiella phage vB_KpnM_BIS47	99.62
	NAD-dependent protein deacetylase of SIR2 family	KPP_11251	Klebsiella phage vB_KpnM_KB57	100
	Uncharacterized domain COG3236 / GTP cyclohydrolase II (EC 3.5.4.25)	KPP_11371	Proteus phage Mydo	90
	Putative exodeoxyribonuclease	KPP_11391	Klebsiella phage vB_KpnM_KB57	97.73
	Phage endonuclease	KPP_11421	Klebsiella phage vB_KpnM_KB57	100
	Putative thymidylate synthase	KPP_11501	Klebsiella phage KNP2	99.4
	Ribonucleotide reductase of class Ia (aerobic), alpha subunit (EC 1.17.4.1)	KPP_11531	Klebsiella phage vB_KpnM_VIK251	99.74
	Ribonucleotide reductase of class Ia (aerobic), beta subunit (EC 1.17.4.1)	KPP_11541	Klebsiella phage vB_KpnM_KB57	100
	PhoH-like protein	KPP_11601	Klebsiella phage vB_KpnM_KB57	100
	Prophage Clp protease-like protein	KPP_11641	Klebsiella phage vB_KpnM_KB57	99.67
	Putative DNA methyltransferase	KPP_11651	Raoultella phage Ro1	100
	CCA tRNA nucleotidyltransferase (EC 2.7.7.72)	KPP_11711	Klebsiella phage vB_KpnM_BIS47	99.52
	Phage-associated homing endonuclease	KPP_12431	Mydovirus KpS8	100
	Putative metal dependent phosphohydrolase	KPP_12491	Klebsiella phage vB_KpnM_KB57	100
	ATPase	KPP_12541	Klebsiella phage vB_KpnM_BIS47	100
DNA replication	DNA helicase, phage-associated	KPP_121	Mydovirus KpS8	100
	Phage DNA primase/helicase	KPP_1111	Klebsiella phage vB_KpnM_BIS47	100
	Phage DNA-directed DNA polymerase (EC 2.7.7.7)	KPP_1131	Klebsiella phage vB_KpnM_BIS47	99.66
	RNA ligase	KPP_11211	Klebsiella phage vB_KpnM_BIS47	100
	DNA ligase	KPP_11261	Klebsiella phage KNP2	99.54
	RNA ligase 2	KPP_12671	Klebsiella phage vB_KpnM_KB57	100

Packaging	Terminase large subunit	KPP_1761	Klebsiella phage vB_KpnM_KB57	100
Lysis	Phage rIIB lysis inhibitor	KPP_111	Klebsiella phage vB_KpnM_KB57	100
	endolysin	KPP_11591	Klebsiella phage vB_KpnM_KB57	100
	Cell wall hydrolase	KPP_12711	Proteus phage Mydo	100
Other	Phage protein	KPP_1541	Klebsiella phage vB_KpnM_KB57	100
	Phage protein	KPP_1611	Klebsiella phage vB_KpnM_KB57	100
